# Soil erosion and corn yield in a cultivated catchment of the Chinese Mollisol region

**DOI:** 10.1371/journal.pone.0221553

**Published:** 2019-10-03

**Authors:** Weige Yang, Xiaocun Zhang, Wei Gong, Yuanyuan Ye, Yongsheng Yang

**Affiliations:** 1 College of Rural Planning and Architectural Engineering, Shangluo University, Shangluo, Shaanxi, China; 2 College of Chemical Engineering and Modern Materials, Shangluo University, Shangluo, Shaanxi, China; 3 Northwest Institute of Plateau Biology, Chinese Academy of Sciences, Xining, China; Centro de Investigacion Cientifica y de Educacion Superior de Ensenada Division de Fisica Aplicada, MEXICO

## Abstract

Evaluation of soil redistribution rates and influence on crop yield in agricultural catchments is very important information, which can provide a scientific basis for arrangement of soil and water conservation measures and sustainable crop production. In recent decades, the soil erosion has greatly aggravated in the Mollisol region of Northeast China due to unreasonable land management, which in turn has reduced crop yield. The objectives of this study were to investigate the spatial distribution of soil redistribution and the relationship between crop yield and soil redistribute at a catchment of the Chinese Mollisol region. A total of 176 soil samples were collected based on a 200 m by 200 m grid and 4 yr of corn (*Zea mays L*.) yields were measured. The ^137^Cs trace technique and Zhang Xinbao’s mass balance model indicated that the soil redistribution rates ranged from −7122.25 to 5471.70 t km^−2^ yr^−1^ and averaged −830.10 t km^−2^ yr^−1^. Soil erosion dominated in the research area. The corn yields for four years ranged from 43.24 to 136.19 kg km^−2^ and averaged 90.42 kg km^−2^. The spatial distribution of soil redistribution rates and corn yield showed a similar ribbon and plaque characteristics at the catchment. An equation between corn yield and soil redistribution rates was fitted and showed that there was a significant negative correlation between corn yield and soil erosion rates, while there was no relationship between the corn yield and soil deposition rates. Therefore, effective soil and water conservation measures are urgently needed to increase crop yield and realize sustainable land-use management.

## Introduction

The Mollisol region in Northeast China is one of China’s most important grain production areas [1]. Approximately half of the country’s corn (*Zea mays*) and a third of its soybean (*Glycine max*) were produced in this region [2–3]. Nevertheless, this region has suffered from serious soil erosion since large-scale reclamation began approximately 100 years ago [4–6]. According to the statistical data reported by the Songliao Water Resources Commission in 2010, 27% of the total territory and 38% of the cultivated area in this region were affected by soil erosion [2]. Consequently, the thickness of the black soils decreased from 60−70 cm in the 1950s to 20−30 cm at present [1, 7, 8]. In some places, the loess parent material was exposed. Soil erosion has also resulted in reduced soil fertility, land degeneration, and crop yield reduction in this region [7]. All these factors represent a considerable threat to China’s food security. Therefore, a better understanding of the spatial of soil erosion rates and its effects on crop yield is important for designing effective soil conservation measures and maintaining soil productivity in this region.

To better understand and manage soil erosion, it is indispensable to accurately estimate soil redistribution rates. Runoff plots, surveying methods and soil erosion models are effective methods to monitor soil erosion, but they are expensive, time-consuming and cannot provide long-term and spatial information of erosion rate [9–11]. The ^137^Cs trace technology overcomes these disadvantages and has been used worldwide in the past few decades [10–14]. For soil erosion based on ^137^Cs technology, numerous publications have focused on at the hill slope scale [14–16], the catchment scale [11, 13, 17–19], and the watershed scale [20, 21]. In the black soil region of Northeastern China, studies on soil erosion using ^137^Cs measurements have also been conducted [22–25]. However, most of the published studies were conducted on the hill slope scale. Only a few studies discussed the rates and patterns of soil redistribution rates in this region using the ^137^Cs technique at the catchment scale [9, 24]. Thus, the use of the ^137^Cs trace technique to study soil redistribution rates and its spatial distribution pattern at the catchment scale is required in this region. On one hand, research on the spatial distribution of soil redistribution could help us to understand the soil mechanism at the catchment scale in the Mollisol region. On the other hand, it can provide the theoretical basis for studying the slope-catchment scale conversion of soil erosion.

Furthermore, the negative effect of soil erosion on agricultural productivity has also become a global problems. In particularly, soil deterioration caused by soil erosion often resulted in decreased crop productivity [26–28]. Over the past fifty years, a number of studies have been performed to investigate the relationship between erosion and crop yields. At present, numerous qualitative studies were mainly conducted in North America, Europe and Australia [7]. In China, soil erosion and crop yields were assessed on the Loess Plateau and in southern China [29–33]. In the Chinese Mollisol region, the effects of soil erosion on crop yield under long-term cultivation have been analyzed on a small field scale over approximately 8 years [28, 30]. Some studies on the relationship between soil erosion and crop yield were conducted over short observation periods [34–36]. There is no information about the relationship between erosion and crop yields on a catchment scale. Therefore, it urgently needs to understanding the response of crop yields to soil redistribution rates on a catchment scale using observational data that have been collected over the years in this region is critical.

The objectives of this study were: (1) to assess the soil redistribution rates applying the ^137^Cs technique and investigate its spatial distribution patterns in the typical Mollisol region of Northeast China. (2) to study the response of the spatial variation of corn yields on soil redistribution rates. and (3) to analyze the relationships between the soil redistribution rates and corn yields.

## Materials and methods

### Ethics statement

The sample points were located on private land. The villagers who owned the land all gave permission to conduct the study at this site. The research sites were not protected in any way and the field studies did not involve endangered or protected species.

### Study area

**Fig 1:**
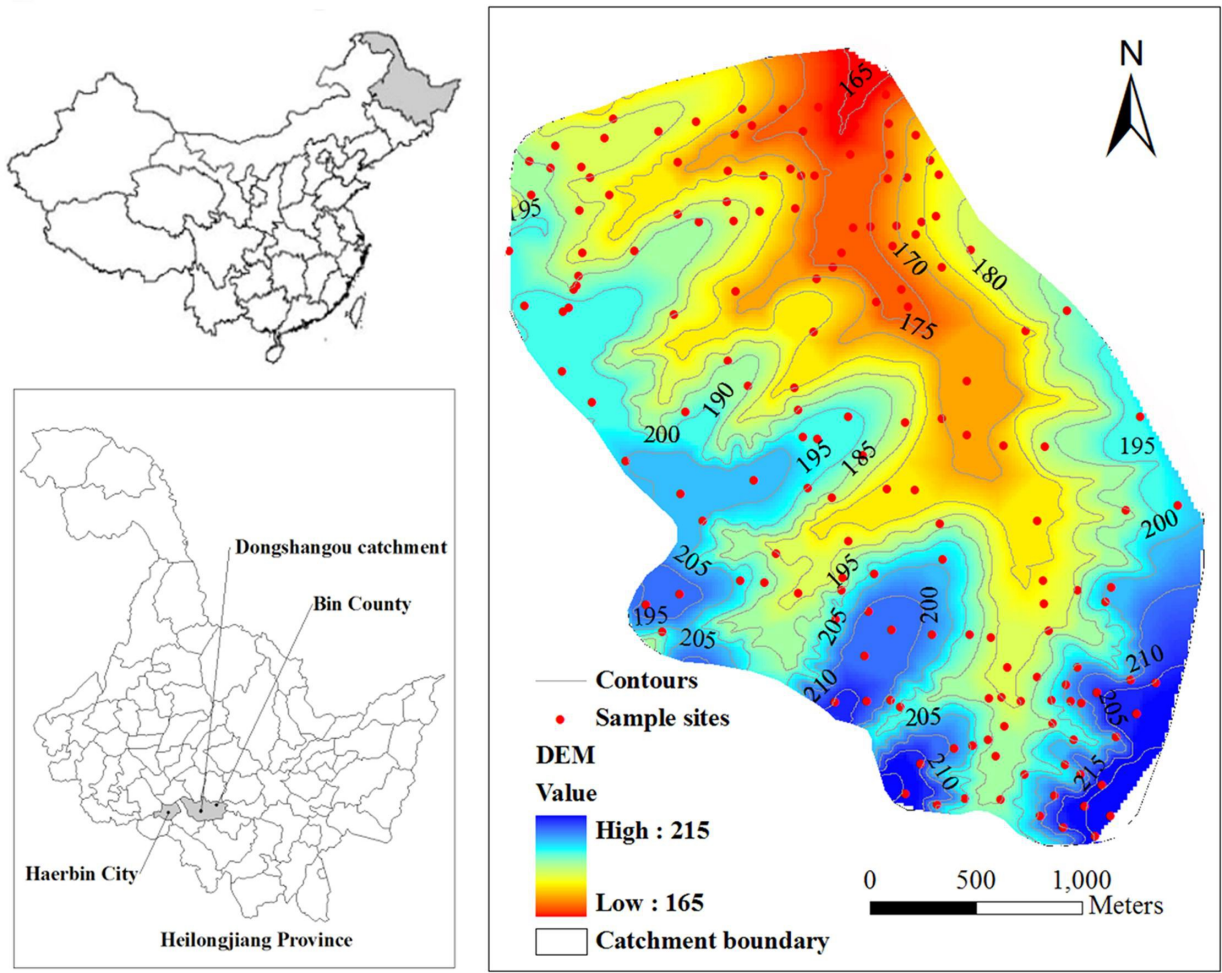
Location of the study area. This figure was made by ArcGIS 9.3 software.

The study was conducted in the Dongshangou small catchment (127°31´–127°34´E, 45°43´– 45°46´E), which located in Bin County in the north of Heilongjiang Province in China (Fig 1). It has an area of 5.52 km^2^. The main geomorphologic characteristic in this region is rolling hilly. The slope length reaches hundreds of meters and the longest can reach thousands of meters. The slope gradient concentrates at 1–7° with a few areas up to 10°. The elevation is relatively low, ranging between 160 and 220 m. The catchment is a semiarid temperate zone with a temperate continental monsoon climate, which is cold and arid in winter and hot and rainy in summer. The mean annual temperature is 3.9°C. The minimum temperature is −29°C in winter, and the depth of frozen soil even reaches 2.4 m. The prevailing wind direction is southeast in summer and northwest in winter. The mean annual precipitation is 548.5 mm, 80% of which is received from June to September. The dominant soil association in this study catchment is classified as Mollisol in the USDA Taxonomy with a clay loam texture [37].

Regarding soil use, the field is devoted to agriculture and is under conventional management. Corn (*Zea mays L*.) has been the dominant crop for several decades, and irrigation is not used in the study area. The field is typically plowed in the late autumn with a cultivator harrow, hoed in spring and ridged with ridges being perpendicular to the slope. The cropland is fallow without any vegetation cover from October to April. The study area is considered to be the representative of the typical Mollisol region given its serious soil erosion, concentrated distribution, and similar weather conditions, agricultural management (i.e., fertilization, tillage regime), crop systems, and productivity levels.

### Soil sampling

First, a detailed field survey, including assessing the topographic, soil erosion and corn yields using a 1:10 000 topographic map and a 1:10 000 land use map of the study catchment, was conducted. Second, the sampling strategy was determined, which was based on a 200 × 200 m grid approach accompanied by the key points sampling method, to assess the spatial distribution of soil redistribution rates and its effects on corn yields. Third, the soil samples were collected after the crop harvest in October 2009 and 2012, and the corn yields (in 2009, 2010, 2012 and 2013 years) at each sampling point were determined at the end of the growth period. Meanwhile, the coordinates and elevation at each sampling site were measured using a Magellan global positioning system tracker (5-m precision).

The soil samples were collected using a 7-cm diameter hand operated core sampler. Soil samples were taken to a depth of 25 cm on the eroded cultivated land and 35 cm on the deposition sites to ensure that the core had penetrated to the full depth of the ^137^Cs profile. From each sampling site, three cores were taken at each corner of a 1 m equilateral triangle and then mixed together to generate a composite sample. Selecting a good reference location for determining the ^137^Cs inventory was critical for this study. After a detailed topographic survey of both the study catchment and its neighboring sites, a reference site with the land use of graveyards nearby the study area was selected [9]. The three eighty-year-old farmers reported that it had not been affected by soil erosion or deposition since 1950. Such a surface condition well satisfied the demands of selecting reference sites described by Quine and Walling [38]. Eight sampling sites were selected randomly on the reference site. In total, there were 176 soil samples for detecting the ^137^Cs activity, including 168 composite samples and 8 reference samples.

At each site, the intact soil cores were sampled using 5 cm (inside diameter) × 5 cm (height) brass rings for bulk density determination.

Afterwards, all soil samples were collected, sealed in plastic bags, and delivered to the laboratory within one week.

### Laboratory analysis

Then, the obtained soil samples were air-dried, and coarse materials, such as gravels and visible pieces of crop residues and roots were removed by hand. The remainder was ground and passed through a 1-mm sieve. Approximately 350 g each sample was weighed for the measurement of ^137^Cs activity.

The samples were sent to the State Key Laboratory of Urban and Regional Ecology Research Center for Eco-Environmental Science, Chinese Academy of Science in Beijing of China, where ^137^Cs activities were measured by gamma-ray spectrometry using a high-resolution, low background, low energy, hyperpure N-type germanium coaxial Y-ray detector (EG&G, ORTEC LOAX HPGe) connected to ORTEC amplifier and multichannel analyzer. To minimize the uncertainties associated with the precision of ^137^Cs measured, the samples in the Marinelli breaker were counted for approximately 28,800 s. This was a sufficient time to obtain a counting precision of ±5% at the 95% confidence level. The ^137^Cs activities in the samples were obtained from the peak area in the part of the spectrum associated with 662 keV [38].

Soil bulk density (BD) was determined by measuring the volume of each original soil sample (100 cm^3^) and its dry mass after oven drying at 105°C.

### Corn yield measurement

Crop yield at each sampling site were measured at three plots each of which was approximately 5 m^2^ (2 m length×2.5 m wide) in size, and with the corresponding soil sampling site was located in the center of the plots or adjacent to the samples site. For each plot, the final crop population and the total amount of straw and grain for each one of the crops were measured. Then, corn samples were harvested at each plot and their fresh weight and dry weight in each plot were measured separately. Corn yield was then calculated based on the dry weight of corn samples in each plot. The yearly corn yield of each sampling site was determined using a mean weight of corn in three sample plots. Yields were averaged across four years to determine the average crop yield (*Y*_*c*_) at each sample site.

### Data analysis

#### Local reference inventory

The average of the ^137^Cs inventory values in the eight samples collected from the reference sites was 2378.40 Bq m^–2^ with a maximum value of 2769.65 Bq m^−2^, a minimum value of 2149.93 Bq m^−2^, a standard deviation of 198.08 Bq m^−2^ and a coefficient of variation (CV) of 8.30%. This value was similar to the reported values of reference inventory values of in some studies [4, 23, 39, 40]. Thus, the established reference ^137^Cs inventory of 2378.40 Bq m^−2^ was regarded as reliable.

#### Calculation of soil redistribution rates

The measurements of ^137^Cs results were originally calculated on a per unit mass basis (Bq kg^−1^) and were then converted into an inventory value (Bq m^−2^) according to the following Eq (1):

$$CPI=\sum_{i=1}^{n}C_{i}\times B_{i}\times D_{i}\times 10^{3}$$ (1)

where *CPI* is ^137^Cs point inventory (Bq m^−2^), *i* is the soil horizon number, *C*_*i*_ is ^137^Cs activity of the *i* th soil horizon (Bq kg^−l^), *B*_*i*_ is bulk density of the *i* th soil horizon (kg m^−3^), and *D*_*i*_ is thickness of the *i* th soil horizon (m).

Soil erosion can be estimated using the simplified mass balance model (SMBM) [41], which has widely been applied to estimate soil redistribution rates from the ^137^ Cs measurements:

$$A=A_{0}\times {\left(1-\frac{h}{H}\right)}^{y-1963}$$ (2)

where *A* is the ^137^Cs inventory at the sampling site (Bq m^−2^), *A*_*0*_ is the local ^137^Cs reference inventory (Bq m^−2^), *h* is the annual soil loss depth (cm), *H* is the plow depth (20 cm), and *y* is the sampling year.

The erosion rate *E* (kg m^−2^ year^−1^) can be expressed as follows:

E=hρ (3)

where *ρ* is the bulk density of the soil (kg m^−3^).

#### Statistical analyses

ArcGIS 9.3 software was used to map spatial distributions of ^137^Cs inventory values, soil redistribution rates and corn yields using the Kriging interpolation method [42]. Linear regression analysis was used to test the correlation between soil distribution rates and corn yields using the software of Sigma Plot 12.0. Statistical analyses were performed using SPSS 16.0 software in the study.

## Results and discussion

### ^137^Cs inventory values and spatial distribution patterns for the catchment

#### ^137^Cs inventory values for the catchment

Descriptive statistics for the activity of ^137^Cs for the 168 soil samples collected from the catchment were provided in Table 1. The ^137^Cs inventory values ranged from 564.11 to 6803.00 Bq m^−2^, with a mean value of 2181.34 Bq m^−2^. For the overall catchment, the coefficient of variation (CV) varied greatly at 47.37%, which was considered to be a medium level according to the classification by Hillel [43]. The medium coefficient of variation value suggested that the ^137^Cs distribution phenomenon with soil particles movement was obvious and emphasized the importance of soil redistribution within the study catchment. Here, 66.67% of the soil samples had lower ^137^Cs inventory values than the reference value of 2379.00 Bq m^−2^ with a mean of 1634.68 Bq m^−2^, implying that the net soil loss occurred at most of the catchment area over the period since the commencement of the ^137^Cs fallout in the middle 1950s. Correspondingly, 33.33% of the ^137^Cs inventory values had higher values than the reference with an average value of 3274.77 Bq m^−2^, indicating that soil deposition have occurred at these sites. Moreover, the coefficient of variation (CV) of ^137^Cs inventory in inventory values less than the reference (29.51%) the same as that in inventory values greater than the reference (29.71%), suggesting that ^137^Cs distribution differentiations with soil particle movement in the erosion and deposition area were similar.

The variability in ^137^Cs inventory values could be considered as a result of the intrinsic heterogeneity of the environment, such as vegetation (acting on interception of rainfall), plant roots, soil micromorphology, soil properties (density, clay content, macropores, cracks, and stones), and disturbance by humans and animals [20]. Sutherland considered that the variability in the physical properties of a grassland soil ranges 15% and 75%; therefore the 47.37% variability in ^137^Cs activity that we found can be considered normal [44].

**Table 1:** ^137^Cs inventory values for the sampling sites.

	Total samples	Inventory values less than the reference	Inventory values greater than the reference
Maximum (Bq m^–2^)	6803.00	2369.11	6803.04
Minimum (Bq m^–2^)	564.14	564.14	2396.29
Mean (Bq m^–2^)	2181.34	1634.68	3274.77
Standard deviation (Bq m^–2^)	1033.40	482.37	973.22
Median (Bq m^–2^)	2062.09	1679.97	2969.37
CV (%)	47.37	29.51	29.72
Skewness	1.55	–0.34	2.09
Number of samples	168	112	56

### ^137^Cs inventory values in the different catchment position

Concerning the variation in ^137^Cs inventory values for the different catchment positions, a clear pattern was observed in the following order: downstream (2290.67 Bq m^−2^) > middle stream (2167.72 Bq m^−2^) > upper stream (2097.37 Bq m^−2^) (Table 2). The ^137^Cs inventory values did not show a clear pattern in the different catchment positions because the ^137^Cs inventory values increase slightly by 8.44% from the upstream to the downstream. The coefficient of variation (CV) of ^137^Cs inventory values in the different catchment positions decreased obviously from the upstream (62.52%) to the midstream (43.93%) and downstream (29.00%). This finding suggested that the ^137^Cs distribution phenomenon was obvious in the entire catchment. Even within the same catchment positions, the variation in ^137^Cs inventory values was considerable, as shown by the CV in excess of 10%.

**Table 2:** ^137^Cs inventory values for the different catchment positions.

	Upstream	Midstream	Downstream
Maximum (Bqm^–2^)	6803.04	4252.18	3932.31
Minimum (Bq m^–2^)	564.14	683.29	1139.11
Mean (Bq m^–2^)	2097.37	2167.72	2290.67
Standard Deviation (Bq m^–2^)	1311.30	952.21	664.21
Median (Bq m^–2^)	1775.77	2150.05	2206.02
CV (%)	62.52	43.93	29.00
Skewness	1.93	0.48	0.47
Number of samples	67	44	57

#### Spatial distribution patterns of ^137^Cs for the catchment

**Fig 2:**
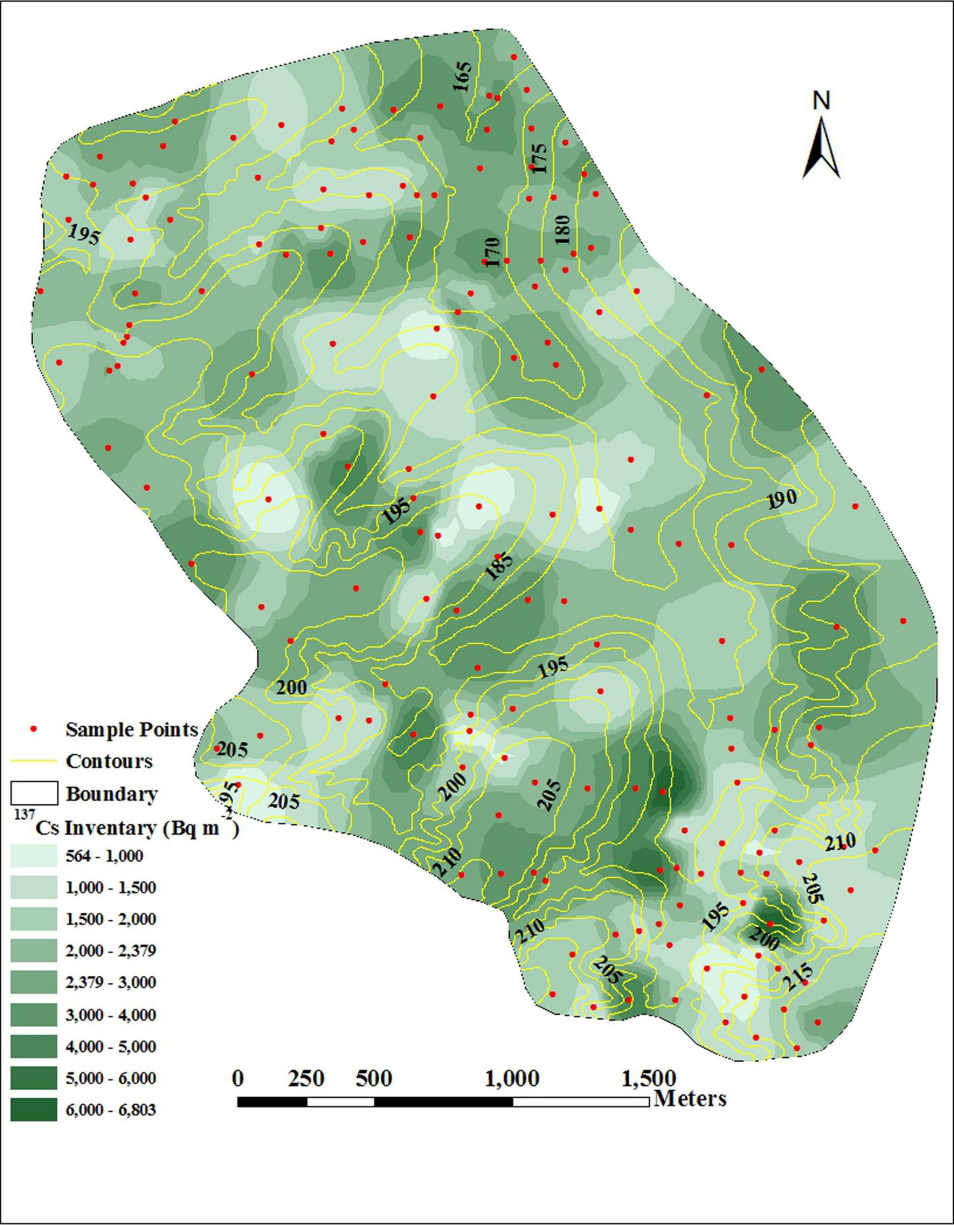
Spatial distribution of ^137^Cs inventory values within the study catchment. This figure was made by ArcGIS 9.3 software.

The spatial distribution of ^137^Cs inventory values in the study catchment was derived by interpolating the values obtained for individual sampling sites using a spatial interpolation procedure (ArcGIS 9.3) constrained by information on the local topography (Fig 2). Overall, the spatial distribution of ^137^Cs inventory values was characterized as ribbon and plaque shapes with high values alternating with low values. The higher ^137^Cs inventory values were located on the slopes and at the catchment outlet; the lower values occurred at the main gully and the ephemeral gullies.

The pattern of ^137^Cs inventory values increased gradually from the gully head to the gully mouth in the main gully. The lowest ^137^Cs inventory values occurred at the gully head. There was evidence of an increase in ^137^Cs inventory values in the middle part of the main gully. At the gully mouth, ^137^Cs inventory values were highest for the main gully.

Moreover, there were some sites where the ^137^Cs inventory values were higher or lower than expected values corresponding to the general pattern (Fig 2), which suggested that ^137^Cs redistribution in these areas has been influenced by the microtopography [9, 45]. The sites where ^137^Cs inventory values were higher than expected were located at the concave area in some slopes. Because the concave shape in this area was cultivated and the slope angles were low, sediment was deposited in the concave area and increased the ^137^Cs inventory values in these areas. The sites where ^137^Cs inventory values were lower than expected were located in the upper slope. Given the slightly convex shape and long tillage in these areas, soils located upslope were transported downward and no soils were replenished. Thus, the ^137^Cs inventory values on the upslope decreased.

### Soil redistribution rates and its spatial distribution patterns

#### Soil redistribution rates in the catchment

Soil redistribution rates which were derived from the ^137^Cs values using the simplified mass balance model for the 168 sampling sites ranged from a maximum erosion rate of −7122.25 to a maximum deposition rate of 5471.70 t km^−2^ yr^−1^ and averaged −830.10 t km^−2^ yr^−1^ (Table 3). The coefficient variation of soil redistribution rates for the overall catchment was high with variability of 272.62%, which was considered to be high levels according to the classification by Hillel [43]. This finding indicated that there was obvious differences in soil redistribution rates existed in the study catchment.

To test the reliability of the observed soil erosion rates value, we compared the value obtained from our estimation with the previous studies in the Mollisol region. The estimated mean erosion rates of the runoff plots during 2003–2004 ranged from 810 to 2820 t km^−2^ yr^−1^ at the Keshan Farm [46]. The estimated soil erosion rates using the ^137^Cs method ranged from −5680 t km^−2^ yr^−1^ to 1 7140 t km^−2^ yr^−1^ with an average of −220 t km^−2^ yr^−1^ and a median of −690 t km^−2^ yr^−1^ at the Heshan Farm, Northwest Heilongjiang Province [4]. Obviously, the averaged erosion rate in this study was similar to these studies.

For the erosion sites, the erosion rates varied from −18.56 to −7122.25 t km^−2^ yr^−1^ with a mean of −2082.37 t km^−2^ yr^−1^. For the deposition area, the deposition rates varied from 38.97 to 5471.70 t km^−2^yr^−1^ with an average of 1423.97 t km^−2^yr^−1^. Moreover, 95.54% of the field was estimated to have exceeded the soil loss tolerance limit of 129 t km^−2^ yr^−1^ which was reported by Duan [47]. It was clear that erosion was the dominant soil redistribution pattern in the study catchment. In addition, according to the soil erosion classification and grading standards [46], which was issued by the Ministry of Water Resources, the dominant erosion classifications were the mild and moderate erosion. Specifically, 53.57% of the 112 sampling sites that experienced erosion were categorized as mild erosion, and 23.46% were categorized as moderate erosion. The unique natural environment and human activity have made the black soil region become the largest potential dangerous area of soil erosion in China [48]. Therefore, even if mild and moderate erosion were the main erosion classifications in the study catchment, the potential hazard cannot be ignored, and effective soil conservation measures are urgently required for sustainable management of soil resources [48].

**Table 3:** Soil loss and deposition rates for the sampling sites in the study catchment.

	Gross samples	Erosion sites	Deposition sites
Maximum (t km^–2^ yr^–1^)	5471.70	–18.56	5471.70
Minimum (t km^–2^ yr^–1^)	–7122.25	–7122.25	38.97
Mean (t km^–2^ yr^–1^)	–830.10	–2082.37	1423.97
Standard deviation (t km^–2^yr^–1^)	2262.99	1633.03	1273.42
Median (t km^–2^ yr^–1^)	–617.46	–1694.40	169.50
CV (%)	272.62	78.42	89.43
Skewness	–0.09	–0.81	1.28
Number of samples	168	112	56

#### Soil redistribution rates in catchment positions

The soil redistribution rates in the different catchment positions also showed a clear pattern in the following order: downstream (–135.01 t km^−2^yr^−1^) > midstream (–938.49 t km^−2^yr^−1^) > upstream (–1350.65 t km^−2^yr^−1^) (Table 4). The soil redistribution rates increased almost 85.61% from the downstream to the midstream and increased 39.08% from the midstream to the upstream. Moreover, the coefficient of variation (CV) of soil redistribution rates in different catchment positions decreased obviously from the upper stream (196.90%) to the downstream (1077.42%). CV values in excess of 100% were classified as intensified variation coefficients, and standard deviations were obviously in excess of median values [43]. Therefore, soil redistribution rates in different stream positions were considerable in the study area.

**Table 4:** Soil loss and deposition rates for the different catchment positions.

	Upstream	Midstream	Downstream
Maximum (t km^−2^yr^−1^)	5471.7	3199.19	2759.19
Minimum (t km^−2^yr^−1^)	–7122.25	–5737.70	–3290.35
Mean (t km^−2^yr^−1^)	–1350.27	–938.49	–135.01
Standard deviation (t km^−2^yr^−1^)	2658.71	2282.45	1454.66
Median (t km^−2^yr^−1^)	–1584.13	–541.27	–83.92
CV (%)	196.90	243.20	1077.42
Skewness	0.47	–0.308	–0.232
Number of samples	67	44	57

#### Spatial distribution patterns of soil redistribution rates for the catchment

**Fig 3:**
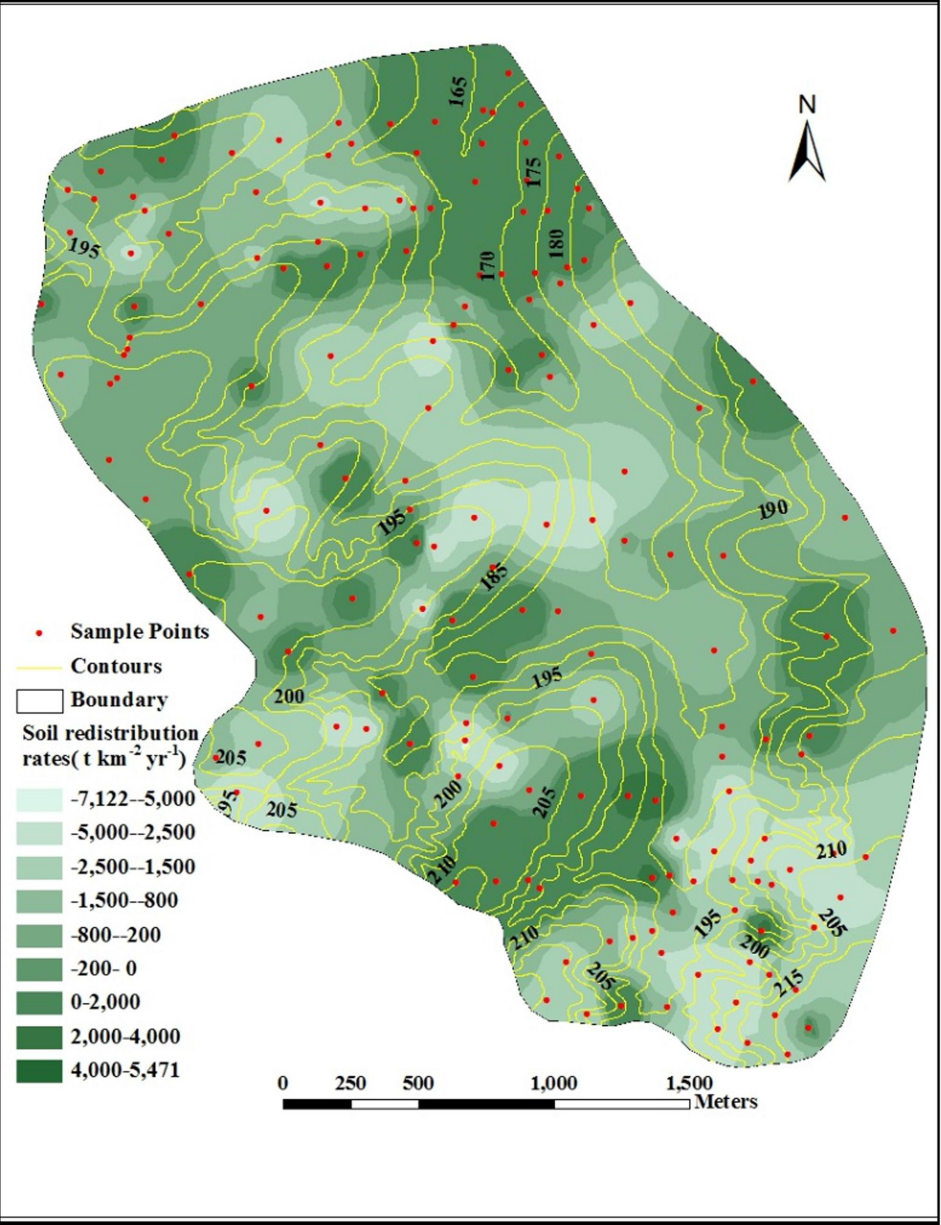
Spatial distribution of soil redistribution rates estimated from ^137^Cs inventory values within the study catchment. This figure was made by ArcGIS 9.3 software.

We used Kriging interpolation to estimate soil redistribution rates at unsampled locations from the available data sites. These estimates were presented as distribution maps in Fig 3. The distribution patterns of soil redistribution rates were the same as that of the ^137^Cs inventory values. The highest soil loss rates were located at the main gully head, which has a convex shape, while the highest soil deposition rates were mainly found at the catchment outlets that were concave in shape.

Overall, Fig 3 demonstrated that the area where erosion occurred was distinctly larger than where soil deposition was found. High erosion rates (>5000 t km^–2^ year^–1^) were found at the main gully head where slope gradients were steepest with the greatest altitude and were coupled with substantial slope lengths in the study catchment. Gully heads were the key position for controlling soil erosion and sediment delivery. This could be accomplished by reducing tillage or straw mulching to reduce runoff and control erosion around gully heads. The high erosion rates (–2500~ –5000 t km^–2^ year^–1^) were found around the branch gullies where slope gradients were relatively high, and many rills’ mouths around them were found. A great quantity of soil material from the upper slopes with surface water flow input the rills and is transported to the branch gullies. Values of –200~ –2500 and 0~ –200 t km^–2^ year^–1^ were found mainly on the slopes where slope gradients were relatively low and there was no opportunity for soil input from upslope given the ridges, which formed the row boundaries. In this case, runoff along with the furrow input the slope rill and the ephemeral or permanent rill gully formed. Rill erosion and tillage erosion were likely the key processes on the slope.

The highest rates of soil accumulation (4000~5471 t km^–2^ year^–1^) were mainly found at the catchment outlet which was the main gully mouth and concave in shape. Values of 200~4000 and 0~2000 t km^–2^ year^–1^ were found mainly on the upper or bottom slopes, which are characterized by the flat topography and large area. Previous studies have shown that the pattern of soil redistribution was characterized by soil erosion from the upper slope positions and deposition at the lower slope positions [17, 49].

### Variability and spatial distribution patterns of corn yield

#### Variability in corn yields for the catchment

To investigate the changes of corn yield in the different catchment positions and the effects of soil erosion and deposition rates on corn yield and to establish the relationships between soil erosion and corn yields, corn yields of the 168 sampling sites were measured for four years. The corn yields for four years ranged from 43.24 to 136.19 kg km^–2^ and averaged 90.42 kg km^–2^ with a middle coefficient of variation of 19.13% (Table 5). This finding revealed there were differences in corn yields in the study catchment. Moreover, mean corn yields were similar to median of corn yields, which showed that corn yields values are less affected by outliers. A middle variability of corn yields was also noted with coefficients of variation of 21.59%, 28.80%, and 23.77% in 2009, 2012, 2013, respectively, and small variability in corn yields of 9.48% in 2010 (Table 5). The maxima of corn yields were 2.45-, 1.62-, 5.80-, and 4.82-fold that of the corresponding minima in 2009, 2010, 2012 and 2013, respectively (Table 5). The large differences in corn yields among the different sampling sites in the same year might be due to the great variation in soil quality, which appeared to be an important factor for corn growth. The mean corn yield in 2013 was 89.57 kg km^–2^, exhibiting a decrease of 1.68% and increases of 3.25% and 0.80% compared to that in 2012, 2009 and 2010, respectively (Table 5). Variation of corn yields in the same field in different years could be mainly attributed to weather conditions, especially rainfall. According to the survey in the study catchment, the rainfall in 2013 and 2012 were significantly greater than that in 2009 and 2008, while the least amount of rain fell in 2010.

**Table 5:** Descriptive statistics of corn yields in 2009, 2010, 2012, and 2013 in the study catchment.

	2009	2010	2012	2013	Average
Maximum (kg km^–2^)	120.87	114.27	147.95	154.22	136.19
Minimum (kg km^–2^)	49.43	70.42	25.52	31.98	43.24
Mean (kg km^–2^)	86.75	89.68	91.11	89.57	90.40
Standard deviation (kg km^–2^)	18.73	8.50	26.24	21.29	17.29
Median (kg km^–2^)	86.86	88.55	91.98	88.67	89.00
CV (%)	21.59	9.48	28.80	23.77	19.13
Skewness	–0.07	0.48	–0.09	0.02	–0.04
Number of samples	36	36	148	149	152

In addition, average of corn yields in the deposition areas were 2.24% greater compared with the erosion sites (Table 6). This finding reveals that soil erosion has reduced corn yields in the study area. The reason was that erosion caused many changes in soil conditions, such as thinning the topsoil, reducing the proportion and stability of large aggregates, increasing soil density, weakening the capacity of retaining water, reducing soil organic matter and nutrient content, and decreasing enzyme activities and microbial activity. All of these factors will further limit crop growth and low crop production. The previous studies noted that wheat grain yields in areas of soil loss were 55% and 41% lower than those in areas of soil accumulation by 5- and 15-tillage operations after intensive tillage, respectively [50]. Given the widely accepted conclusion that crop yields are reduced by more than 10% with soil erosion, crops were reduced by 50% in plots with serious erosion [51].

**Table 6:** Corn yields in soil erosion area and deposition area in the study catchment.

Item	Erosion site	Deposition site
Maximum (kg km^–2^)	136.19	130.33
Minimum (kg km^–2^)	46.98	43.24
Mean (kg km^–2^)	89.65	91.66
Standard deviation (kg km^–2^)	17.29	17.38
Median (kg km^–2^)	88.07	89.92
CV (%)	19.29	18.96
Skewness	0.10	–0.28
Number of samples	95	57

#### Corn yields at catchment positions

**Fig 4:**
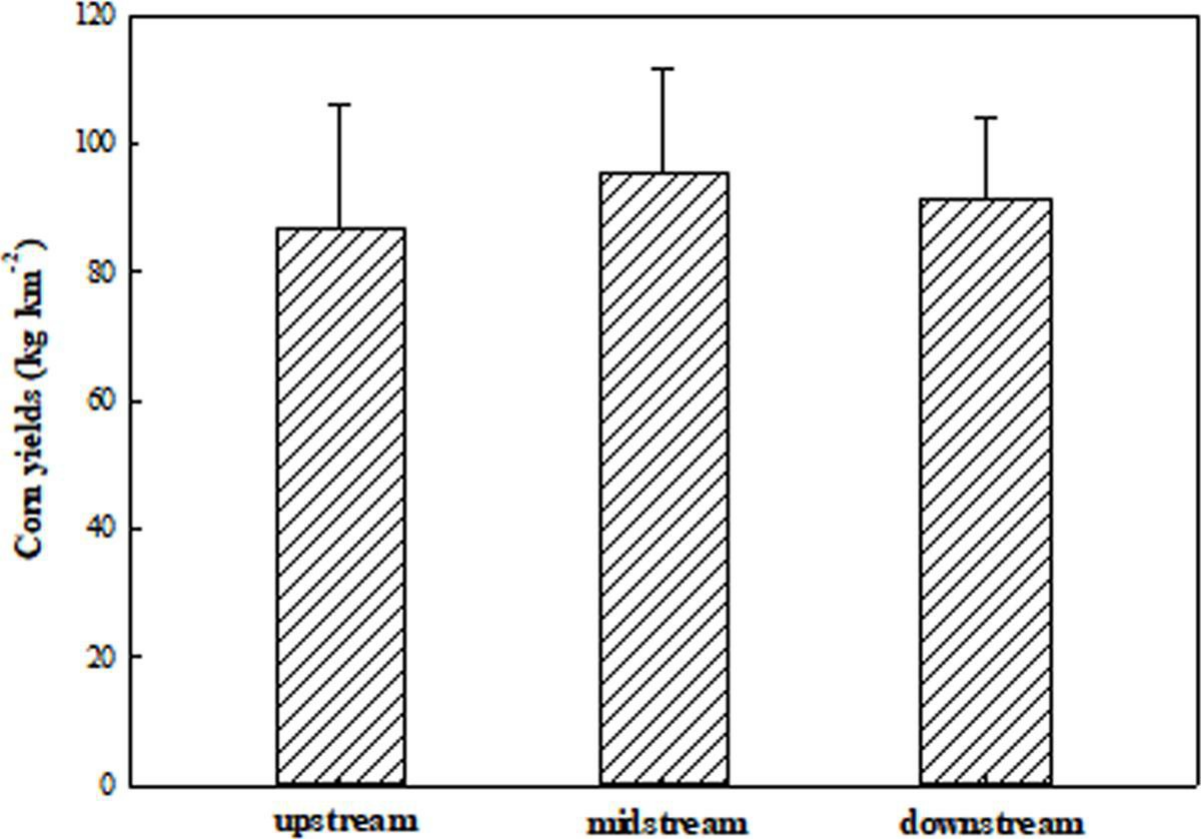
Corn yields for the different catchment positions.

The corn yields change in the study catchment (Fig 4) occurred in the following order: midstream> downstream> upstream. The average corn yields in the upstream were 23.18% and 10.89% lower than that in the midstream and the downstream, respectively. Comparing the soil redistribution rates and corn yields in the catchment positions, the highest erosion rates corresponded to the lowest corn yields in the upstream position, but there was no one-to-one corresponding relationship in the midstream and downstream positions. This may be attributed to the fact that the variation in corn yield depends on not only soil redistribution rates but also other factors, such as soil quality, water, air and soil management factors.

#### Spatial distribution of corn yields

**Fig 5:**
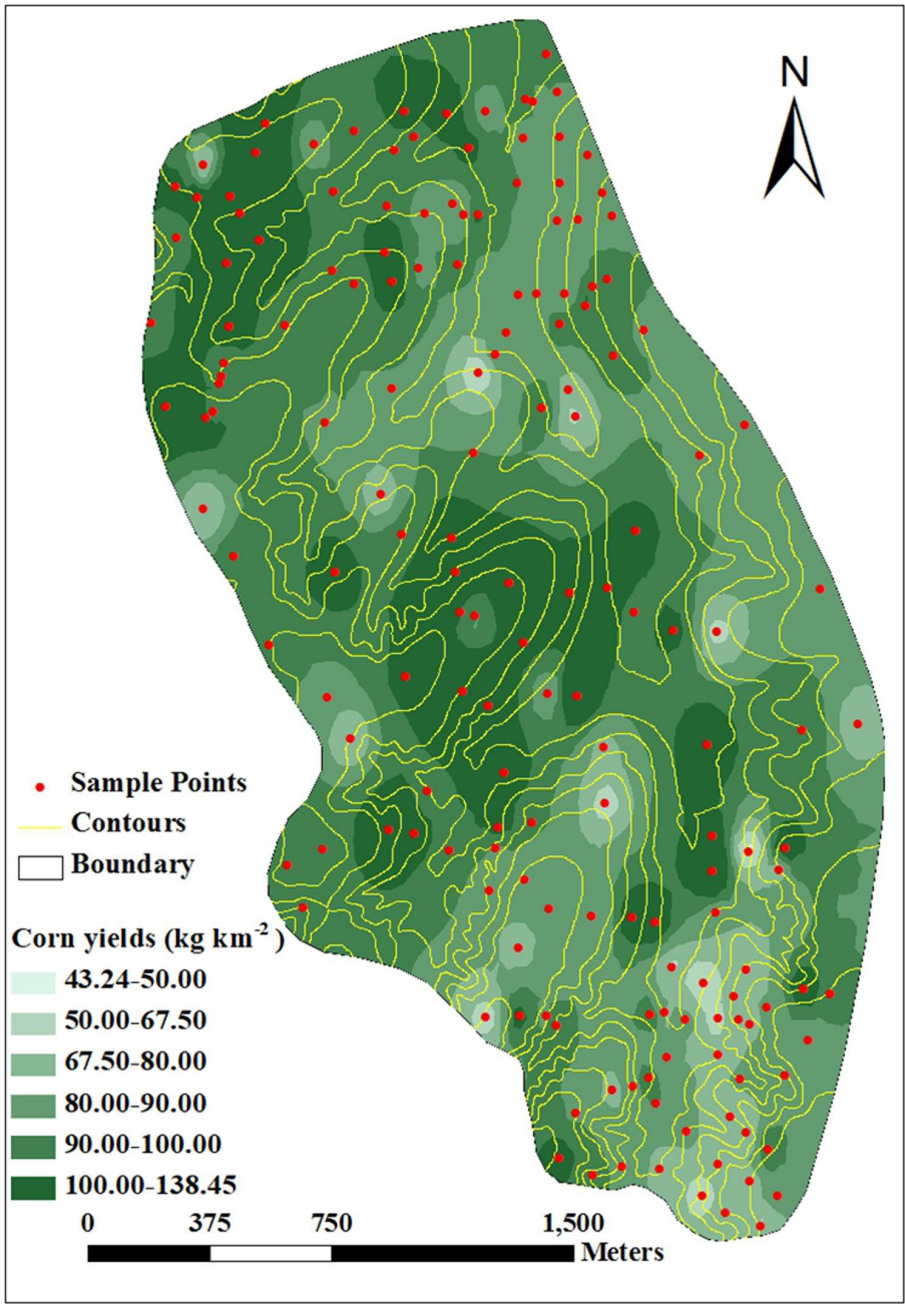
Spatial distribution of corn yields in the study catchment. This figure was made by ArcGIS 9.3 software.

We also used the spatial interpolation procedure to estimate crop yields in the study catchment at unsampled locations from the available data sites. The results of this spatial interpolation process are shown in Fig 5. The spatial distribution of corn yields presented ribbon and plaque shapes in the study area. Furthermore, low corn yields were mainly located in the south of the study catchment, and high corn yields were found in the middle and north. Crop yields in the main and branch gullies were less than that in the slope.

### Correlation between corn yields and soil redistribution rates

#### Correlation between soil deposition rates and corn yields

The correlation between soil deposition rates and corn yields was analyzed. The result showed that no significant correlations existed between soil deposition rates and corn yields in this study catchment. This finding indicated that the relationship between soil deposition rates and crop production was not a simple one in this study catchment; therefore, other factors must be considered. Soil physical, chemical, and biological properties; landscape position; the sloping topography; microtopography; and other factors influence corn yield in the deposition area.

#### Correlation between soil erosion rates and corn yields

**Fig 6:**
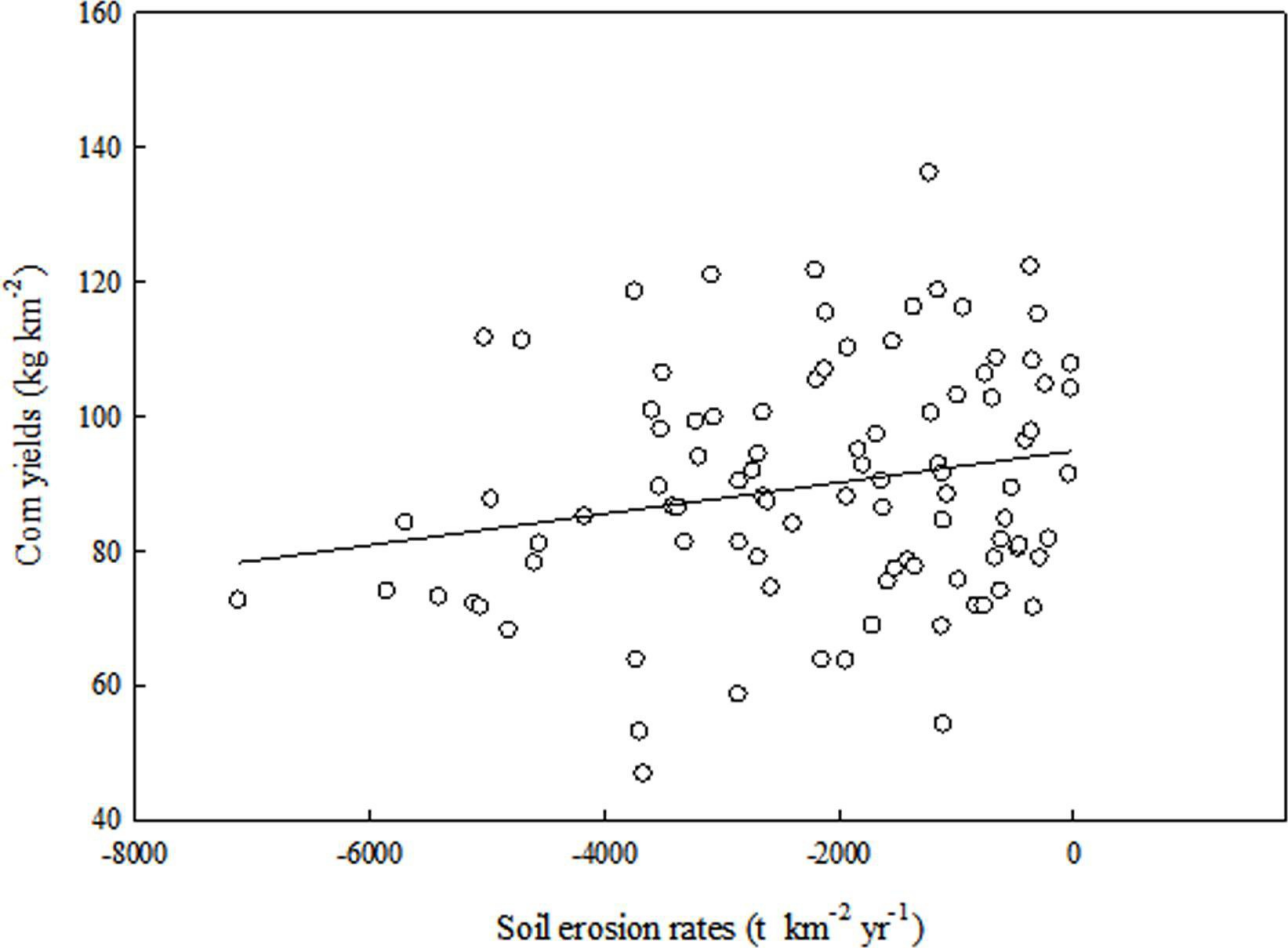
Relationship between soil erosion rates and corn yields.

Fig 6 showed the correlation between corn yield and soil erosion rates, based on the following equation:

$$Y_{c}=94.93+0.0023\;X\;(n=95,r^{2}=0.21;p=0.04<0.05)$$ (4)

where *Y*_*c*_ represents the corn yields (kg km^–2^), and *X* represents the erosion rates (t km^–2^ yr^–1^).

The results indicated that corn yields were significantly negative correlated with soil erosion rates in the study area (Fig 6), confirming that corn yield was indeed affected by soil erosion. This linear regression equation was also consistent with studies by Lar [52] and Hurni [53]. Other authors used the exponential and S-shaped functions to qualify the relation between erosion and crop yield [54–57]. Overall, soil erosion showed a negative effect on crop yield and affected its spatial distribution.

## Conclusions

To evaluate the relationship between crop yield and soil erosion in the typical Mollisol region of Northeast China, the ^137^Cs technique was used to estimate soil redistribution rates for a 5.52-km^-2^ catchment and corn yields for four years. The results from the simplified mass balance model showed that the averaged soil redistribution rate was −830.10 t km^−2^ yr^−1^ for 168 sampling sites. Obviously, erosion dominated in the study catchment. The percentages of samples sites that exhibited mild and moderate erosion were 53.57% and 23.46%, respectively. The spatial distribution of soil redistribution rates revealed that the highest soil loss rates were located at the main gully head, while the highest soil deposition rates were mainly found at the catchment outlet. The spatial distribution of crop yield at the catchment corresponded to the distribution of soil erosion to a certain extent. Moreover, this study demonstrated no significant correlation between soil deposition rates and corn yields, while significant negative relationships were noted between soil erosion rates and crop yields, suggesting that the smaller crop yields occurred where the more serious soil loss was noted. Therefore, soil conservation measures are urgently required to reduce soil erosion for the purposes of sustainable agriculture, such as soil conservation tillage, including remaining residual and straw in the field, mulch cover and no-till.

## Supporting information

S1 Data (ZIP)
